# Improved Manual Annotation of EEG Signals through Convolutional Neural Network Guidance

**DOI:** 10.1523/ENEURO.0160-22.2022

**Published:** 2022-09-27

**Authors:** Marina Diachenko, Simon J. Houtman, Erika L. Juarez-Martinez, Jennifer R. Ramautar, Robin Weiler, Huibert D. Mansvelder, Hilgo Bruining, Peter Bloem, Klaus Linkenkaer-Hansen

**Affiliations:** 1 Department of Integrative Neurophysiology, Center for Neurogenomics and Cognitive Research (CNCR), Amsterdam Neuroscience, Vrije Universiteit Amsterdam, Amsterdam 1081 HV, The Netherlands; 2 Child and Adolescent Psychiatry and Psychosocial Care, Emma Children’s Hospital, Amsterdam University Medical Centers, Amsterdam 1105 AZ, The Netherlands; 3 N=You Neurodevelopmental Precision Center, Amsterdam Neuroscience, Amsterdam Reproduction and Development, Amsterdam University Medical Centers, Amsterdam 1105 AZ, The Netherlands; 4 Levvel, Center for Child and Adolescent Psychiatry, Amsterdam 1105 AZ, The Netherlands; 5 Informatics Institute, Vrije Universiteit Amsterdam, Amsterdam 1081 HV, The Netherlands

**Keywords:** artifact detection, convolutional neural networks, deep learning, digital signal processing, EEG

## Abstract

The development of validated algorithms for automated handling of artifacts is essential for reliable and fast processing of EEG signals. Recently, there have been methodological advances in designing machine-learning algorithms to improve artifact detection of trained professionals who usually meticulously inspect and manually annotate EEG signals. However, validation of these methods is hindered by the lack of a gold standard as data are mostly private and data annotation is time consuming and error prone. In the effort to circumvent these issues, we propose an iterative learning model to speed up and reduce errors of manual annotation of EEG. We use a convolutional neural network (CNN) to train on expert-annotated eyes-open and eyes-closed resting-state EEG data from typically developing children (*n* = 30) and children with neurodevelopmental disorders (*n* = 141). To overcome the circular reasoning of aiming to develop a new algorithm and benchmarking to a manually-annotated gold standard, we instead aim to improve the gold standard by revising the portion of the data that was incorrectly learned by the network. When blindly presented with the selected signals for re-assessment (23% of the data), the two independent expert-annotators changed the annotation in 25% of the cases. Subsequently, the network was trained on the expert-revised gold standard, which resulted in improved separation between artifacts and nonartifacts as well as an increase in balanced accuracy from 74% to 80% and precision from 59% to 76%. These results show that CNNs are promising to enhance manual annotation of EEG artifacts and can be improved further with better gold-standard data.

## Visual Abstract

**Figure:**
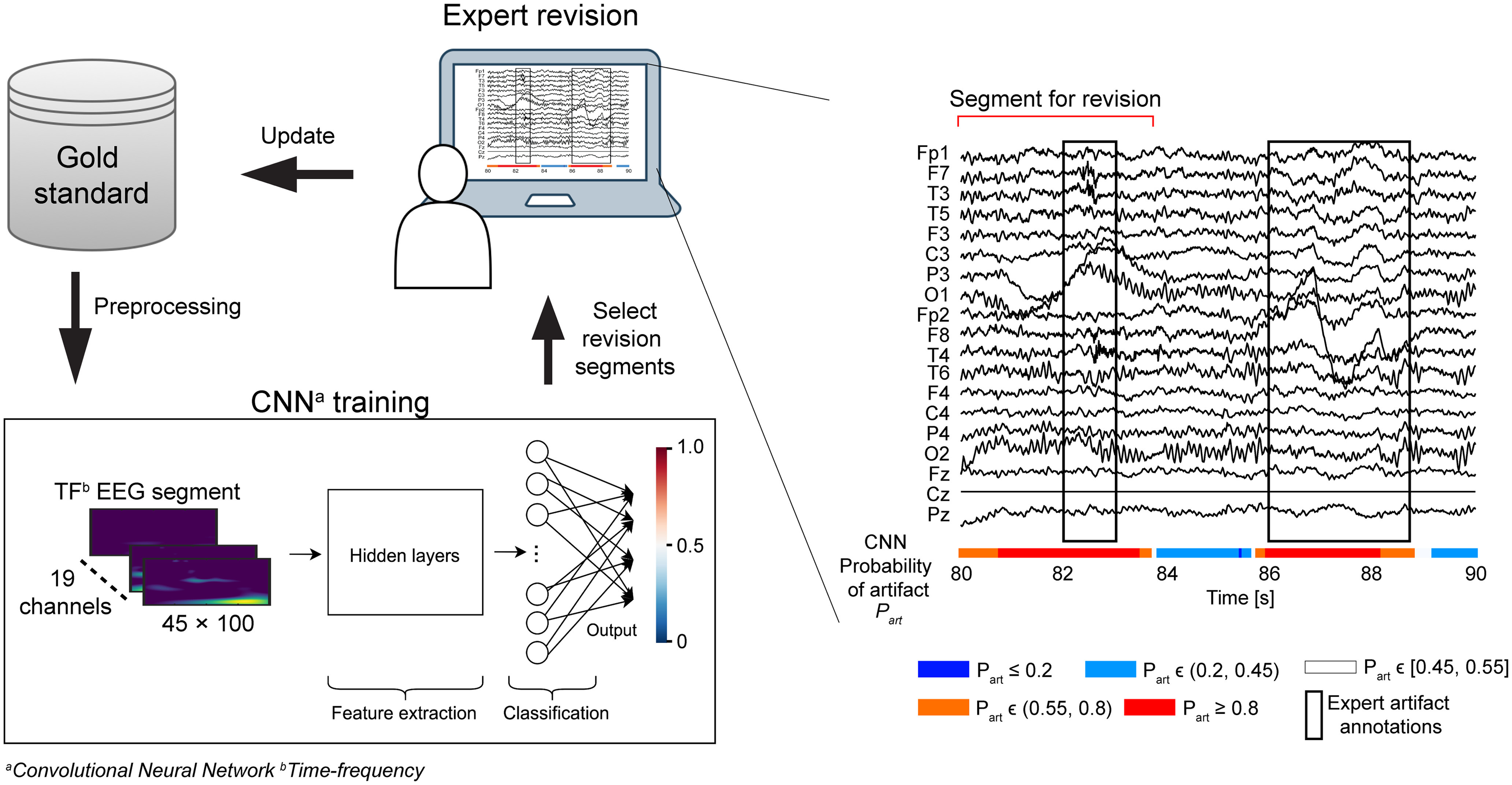

## Significance Statement

Manual annotation of artifacts in EEGs remains the gold standard in research and clinic but is time consuming and prone to human oversight. Here, we introduce a convolutional neural network (CNN) to increase the speed and accuracy of manual annotation of EEG artifacts. We highlight the possibility of using active learning to iteratively improve both the model and the gold standard. With our method, it is possible to vary the decision probability threshold and control the portion of the data that can be labeled automatically by the model or that would require expert judgment. We expect that our new approach will speed up EEG processing and facilitate reliable data analysis in neurodevelopmental disorders.

## Introduction

EEG recordings contain a mix of complex signals coming from both neuronal and non-neuronal sources. The latter sources produce artifacts which, in turn, can have physiological or nonphysiological origins such as muscle activity or electrode movement, respectively. Artifacts are commonly manually identified and removed from the data before EEG signals are analyzed further. The quality and reliability of data analysis ultimately depend on the definition of artifacts, subjective decisions, concentration of the professional who preprocesses the data, and subsequently the resulting quality of the preprocessed signals. The annotation procedure is time consuming, complicating the assessments of large datasets or delaying the analysis of noisy EEG recordings in certain patient populations, such as in children with neurodevelopmental disorders who may find it difficult to sit still during the recording. Thus, reliable automated artifact detection methods would be an asset; however, a consensus is lacking on how to identify the large diversity of artifacts in a reliable manner, and manual annotation remains a gold standard (Urigüen and Garcia-Zapirain, 2015).

Several advanced algorithms have been developed for automated EEG preprocessing of artifacts. These algorithms are built on signal-processing techniques such as regression (Anderer et al., 1999; Croft and Barry, 2000), independent component analysis (ICA; Bell and Sejnowski, 1995; Delorme et al., 2007; Vigario and Oja, 2008), or a wavelet transform (A Cohen and Kovačević, 1996; Unser and Aldroubi, 1996). Automation is mainly achieved through channel referencing (Schlögl et al., 2007), by applying various thresholding mechanisms (Castellanos and Makarov, 2006; Gao et al., 2010; Nolan et al., 2010; Mognon et al., 2011; Akhtar et al., 2012; Islam and Tcheslavski, 2016; Jas et al., 2017), or using feature extraction followed by classification with conventional machine-learning algorithms such as support vector machines (Shoker et al., 2005; Halder et al., 2007; Shao et al., 2009; Gabard-Durnam et al., 2018; Sai et al., 2018). In recent years, deep-learning algorithms have gained popularity to address EEG signal denoising (Wang et al., 2018; B Yang et al., 2018; Craik et al., 2019; Pion-Tonachini et al., 2019; Roy et al., 2019; Sun et al., 2020; Boudaya et al., 2022; Jurczak et al., 2022; Liu et al., 2022), providing a more flexible solution than traditional methods by taking advantage of end-to-end learning, i.e., using a single model to act as both feature extractor and classifier. For example, because of hierarchical feature learning, convolutional neural networks (CNNs; LeCun et al., 1989, 1998, 2010, 2015) can recognize complex patterns from minimally preprocessed data. This strength may be applicable to discriminate complex EEG patterns produced by the brain from various nonbrain artifacts. Developing these methods requires big datasets, and recent large-scale open-source data-sharing initiatives (Harati et al., 2014; Cavanagh et al., 2017) are a great source of EEG data. Nonetheless, openly accessible annotated datasets are scarce (Hamid et al., 2020; Buckwalter et al., 2021; Zhang et al., 2021), and validation of artifact-detection approaches is problematic as no gold-standard and standardized benchmarks are currently available.

Sometimes active-learning approaches are used to generate more labeled data (Settles, 2009; Lawhern et al., 2015; Sebek et al., 2019). Typically, such approaches start with a model trained on a small labeled training set and use expert knowledge for manual labeling of the most useful (i.e., least confident) examples to add them into the training set and iteratively repeat the procedure. However, the amount of data may be not enough to start with as deep-learning methods need big-size datasets to be sufficiently trained. Moreover, such methods operate under an assumption that a ground truth is reliable, which is not always the case.

In this proof-of-concept study, we propose an iterative deep-learning-based approach that could accelerate and increase the quality of manual annotation of artifacts in resting-state multichannel EEG recordings and improve gold-standard signal data that would be suitable for the development and validation of artifact detection and removal techniques. We hypothesize that CNNs trained on expert-annotated EEG data can be used to revise and improve the gold standard, which, in turn, can be used to improve the model. We also argue that automatic preprocessing algorithms are currently unable to fully replace humans in the decision-making process but should rather be used to speed up and reduce errors of manual EEG annotation. Using such a decision-support system may be reciprocally beneficial, as both the human and the system would actively learn from each other and improve their performance. Thus, we intent to integrate this approach into a toolbox to facilitate annotation of EEG signals, its further testing, as well as data curation and sharing.

## Materials and Methods

### Definitions

Inconsistencies between the definition of artifacts from task to task or expert to expert are among factors that complicate standardization of benchmarks and validation of methods. Here, a common convention of defining artifacts as any nonbrain-arising activity reflected on EEG traces was used. The task of artifact classification was defined as follows: “Given a multichannel EEG pattern, determine if it contains an artifact.”

To avoid ambiguity when using terminologies of EEG and machine learning which share a few identical words with different meanings, clarifications and definitions are provided throughout this paper.

### Task formulation

Mathematically, the task is formulated in the following way. Given a dataset of EEG segments obtained from minimally preprocessed recordings measured on different individuals, it can be written: $D=\{\left(X^{\left(1\right)},y^{\left(1\right)}\right),\left(X^{\left(2\right)},y^{\left(2\right)}\right),...,(X^{\left(N\right)},y^{\left(N\right)})\}$, where $X^{\left(i\right)}$ denotes the *i*-th EEG segment, $y^{\left(i\right)}$ is its class label, and *N* is the total number of segments in the dataset. The input structure $X^{\left(i\right)}$ is a tensor with $C_{in}\times m\times n$ dimensions which describes the *i*-th EEG segment. Here, $C_{in}$ indicates the number of channels (i.e., the size of a vector of features associated with each pixel) in the input image, m is the image height and n is the image width. In general, representation of EEG can vary and depends on the desired input formulation, goal, and algorithm. Signal values (discretized voltage fluctuations) and images [derived from time-frequency (TF) analysis] are the most common representations used (Craik et al., 2019; Roy et al., 2019). In this project, we define input as TF images which captured the power spectral density patterns of signal snapshots (segments) and corresponded to a distinct class. Dimensions $C_{in}\times m\times n$, then, correspond to EEG channels × frequencies × time. In the case of binary classification, $y^{\left(i\right)}\in L=\{l_{1}="artifact",l_{2}="non-artifact"\}$, where L is a set of two class labels, and i=1...N.

The goal of training a CNN is to find a set of good parameters θ such that the trained network can take a new previously unseen EEG segment $X^{\left(j\right)}$ and assign the correct class label $y^{\left(j\right)}$ to it: $f\left(X^{\left(j\right)},\text{θ}\right):{\mathbb{R}}^{C_{in}\times m\times n}\to L$, where ℝ is bound to [0,1].

### Data

#### Description

EEG measurements were collected from two ongoing studies with identical EEG measurement protocols [SPACE (Sensory Processing in Autism and Childhood Epilepsy) and BAMBI (Bumetanide in Autism Medication and Biomarker, Eudra-CT 2014-001560-35)]. The studies were conducted in accordance with the guidelines and regulations approved by the respective ethical committee and in compliance with the provisions of the declaration of Helsinki and Good Clinical Practice, and in accordance with the Medical Research Involving Human Subjects Act (WMO). Human subjects were recruited at the Brain Center Rudolf Magnus at the University Medical Center (UMC) Utrecht. Written informed consent was received from the participants or their legal guardians before inclusion in the studies. The dataset comprised recordings of 121 children with autism spectrum disorder (ASD), 20 with epilepsy (EP), and 30 with typical development (TD) aged 7–16 years, with 114 males and 57 females. Signals were recorded using 64-channel BioSemi 10–20 layout caps at 2048-Hz sampling rate during 3–5 min of eyes-closed or eyes-open rest (ECR and EOR, respectively). A total of 340 EEG recordings were available. Manual annotation of artifacts in this dataset was performed by a medical expert with training in clinical EEG (neurophysiology and EP) using information from the 64 channels. Before annotation, the data were bandpass-filtered in the range of 0.5–45 Hz (we used the same range when preprocessing the data as described below, Minimal preprocessing). Cz was used as the reference electrode to perform the annotations. Signals were scrolled through in windows of 10 s. Artifacts included physiologic ones: ocular, cardiac/pulse, glossokinetic, muscle and movement artifacts, and nonphysiologic ones such as electrode detachment (electrode “pop” and bad channels). Artifact definitions include (but are not limited to): activity or waveform confined to a single channel, high voltage, low (<1 Hz) or very high (>70 Hz) frequency fluctuations, double or triple phase reversals and periodic patterns. For a comprehensive review on artifact definition, localization, and atlas see Lüders and Noachtar (2000), Kellaway (2003), Abou Khalil and Misulis (2006), Tatum et al. (2011), Tatum (2014), and Britton et al. (2016). It should be noted that this annotation was not performed for this particular study (i.e., to detect artifacts in particular), but for a clinical research project with the mindset of keeping as much data as possible (Bruining et al., 2020)

#### Minimal preprocessing

EEG recordings were preprocessed using *MNE Python* (Gramfort et al., 2013). Signals were bandpass filtered between 0.5 and 45 Hz using a FIR-filter with a Hamming window and a transition bandwidth of 0.5 Hz at the low cutoff frequency and 11.25 Hz at the high cutoff frequency. The length of the filter was determined from the shortest of the transition bandwidths ( TB=0.5 Hz) and the sampling rate ( SR=2048 Hz) as (3.3⋅SR)/TB and rounded up to the nearest even integer. Bad channels were interpolated using spherical spline interpolation. Recordings were re-referenced using average reference, and 19 standard EEG channels were selected: Fp1, F7, T3, T5, F3, C3, P3, O1, Fp2, F8, T4, T6, F4, C4, P4, O_2_, Fz, Cz, Pz. The selection was limited to 19 standard channels for several reasons. First, there are numerous different low-density and high-density EEG-channel layouts, and many of these layouts are extensions to the standard 10–20 system. Thus, this selection allows to use the model for EEGs recorded with other channel-layout caps. Second, neighboring electrodes are usually highly correlated in high-density-layout caps and will not carry new information. Finally, it will help to reduce computation costs associated with training the model.

#### EEG segmentation and class assignment

Signal segmentation was done using a sliding window of 1 s with 50% overlap between consecutive windows. These values were optimal to enable detection of both slow and fast EEG patterns. A segment was assigned to the nonartifact class if there were no intersections with any of the expert-annotated EEG intervals of artifacts. A segment was assigned to the artifact class if the length of the intersection was at least 0.1 s, or less in the case when the duration of the interval itself was less or equal than 0.1 s. A segment was ignored if the intersection length was less than and the annotation interval duration was >0.1 s. The number of generated segments for each class is specified in Table 1.

**Table 1:** Summary of the EEG data

Group	Number of subjects	Total length of signal (s)	Total length of annotated intervals of artifacts (s)	Number of artifact segments	Number of nonartifact segments
ASD^*a*^ EP^*b*^ TD^*c*^ Total	121 20 30 171	62,790 10,193 16,829 89,812	17,195 1824 2574 21,593	40,265 4332 6209 50,806	82,803 15,780 26,921 125,504

Artifact and nonartifact segments are standardized windows of fixed length of 1 s with 50% overlap between consecutive windows.

a Autism spectrum disorder.

b Epilepsy.

c Typically developing.

#### TF inputs

**Figure 1.**
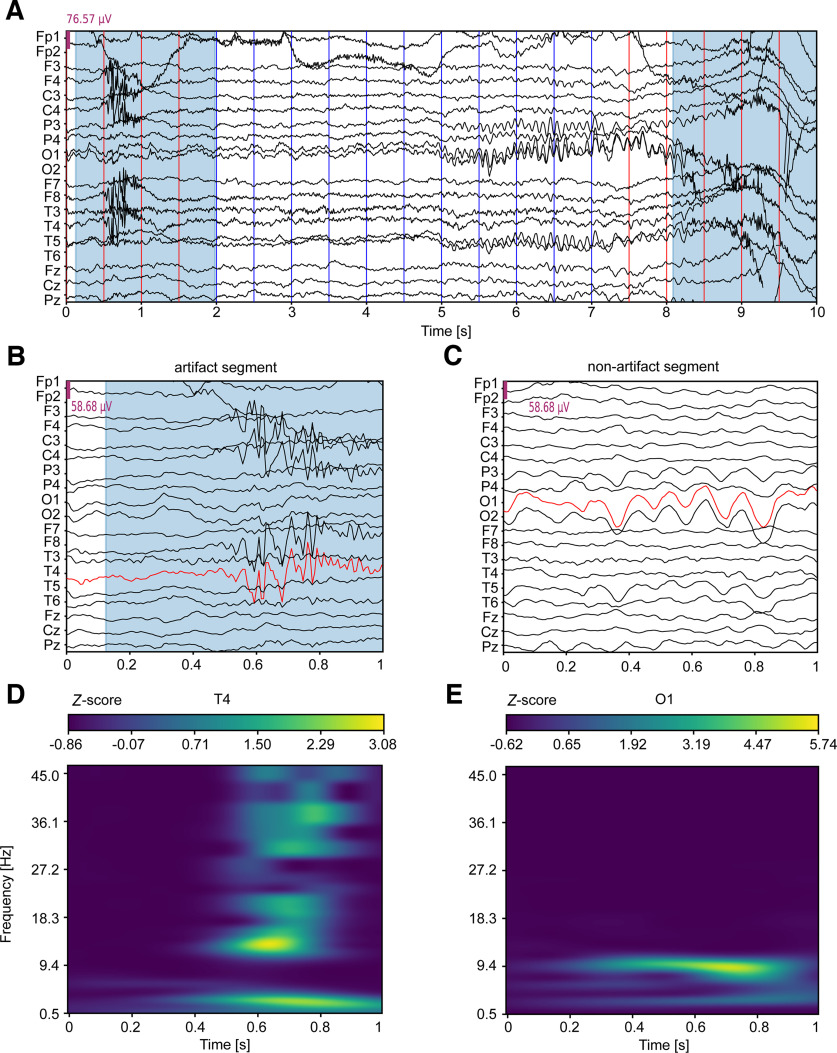
Examples of EEG artifact and nonartifact segments. ***A***, 10 s of an EEG recording. Traces for 19 EEG channels specified on the *y*-axis are shown. Blue shaded regions show manually-annotated artifacts. Red and blue vertical lines indicate onsets (every 0.5 s) of artifact and nonartifact overlapping EEG segments, respectively. ***B***, EEG segment containing an artifact and (***C***) EEG segment that does not contain an artifact. Both show preprocessed EEG signals of 1 s each. ***D***, ***E***, Time-frequency (TF) representations for the EEG channels highlighted in red in ***B*** and ***C***, respectively. The color codes *Z*-score normalized TF power values.

Each 1-s 19-channel EEG segment was transformed into TF domain using complex wavelet convolution. Morlet wavelets were constructed over 45 logarithmically-spaced frequency bins in the range of 0.5–45 Hz. The time resolution parameter as a function of frequency was specified as a logarithmically-spaced vector between 1.2 and 0.2 s, i.e., increasing resolution for higher frequencies (MX Cohen, 2019). Wavelet convolution was performed per EEG channel, and the convolution output was resampled from 2048 to 100 Hz along the time axis. This resulted in a 19×45×100 tensor for each segment. Values were normalized using *Z*-score normalization (with zero mean and unit variance) across all channels. Examples of EEG segments are shown in Figure 1.

### Model

#### Network architecture and structural hyperparameters

In our study, we opted for CNNs as they are known to work well with images which, in our case, are TF representations of EEG signal snapshots. The CNN architecture was created using three convolutional layers with rectified linear unit (ReLU) activation (a function that introduces nonlinearity; LeCun et al., 2015) and one fully connected layer with a softmax (a normalized exponential function; Goodfellow et al., 2016). In addition, max-pooling (a technique to reduce dimensionality of the input) after each convolutional layer with ReLU was introduced in the design. Convolution in the first and second convolutional layers was done per group, i.e., separately for each of the EEG channels in the first layer and for the convolution output of each of the channels in the second convolutional layer. Table 2 provides a summary of the network’s layers, hyperparameters, input and output sizes of each layer as well as the number of learnable parameters.

**Table 2:** Summary of hyperparameters, input/output sizes, and learnable parameters of the CNN architecture used for training

Layer	Input size	Filters	Groups	Kernel	Output size	Parameters
Input (TF image with 19 channels)	19 × 45 × 100	-	-	-	-	-
Convolutional (ReLU)	19 × 45 × 100	50	19	1 × 5 (stride 1 × 1)	950 × 45 × 96	5700
Max-pooling	950 × 45 × 96	-	-	1 × 2 × 2 (stride 1 × 2 × 2)	950 × 22 × 48	-
Convolutional (ReLU)	950 × 22 × 48	100	50	5 × 5 (stride 1 × 1)	1900 × 18 × 44	904,400
Max-pooling	1900 × 18 × 44	-	-	1 × 2 × 2 (stride 1 × 2 × 2)	1900 × 9 × 22	-
Convolutional (ReLU)	1900 × 9 × 22	150	-	3 × 3 (stride 1 × 1)	150 × 7 × 20	2,565,150
Max-pooling	150 × 7 × 20	-	-	1 × 1 × 1 (stride 1 × 1 × 1)	150 × 7 × 20	-
FC (linear)	21,000	-	-	-	2	42,000
Softmax	2	-	-	-	2	-
Output (class distribution)	-	-	-	-	2	-

Here, the input in the first layer is a TF image with 19 channels corresponding to 19 EEG channels, and the output of the last layer is a class probability distribution. No padding (i.e., an area of values, usually zeros, that can be added to the borders of the input, increasing its size) was used. For filter weights, we used Kaiming uniform initialization (He et al., 2015), a default in *PyTorch* implementation of the convolutional layers. Kernel, a two-dimensional matrix of weights that is convolved over the input (in convolutional layers). Multiple kernels form a filter. In pooling layers, there are no filters, and a kernel “summarizes” input values during each sliding step. Stride: a sliding step of a kernel in convolution or pooling. Max-pooling: dimension reduction involving replacing a patch of n×n pixels in the input with a single pixel containing the maximum value from among the pixels of the patch. For multiple dimensions, sizes are of shape channels × frequencies × time.

#### Hyperparameters related to learning

Training was performed using mini-batches. A mini-batch (which we will call a batch thereafter) is a fixed-size group of examples/instances (i.e., single objects from a training, validation, or test set that are supplied to a deep-learning network as input) that is provided to the network during one iteration. In our case, instances are 1-s EEG segments. Based on the results from small-scale experimental runs, a batch size of 64 and the learning rate of 1 × 10^−4^ were selected for full-scale network training and evaluation. An averaged stochastic gradient descent (ASGD) optimizer from *PyTorch* (Paszke et al., 2019) to accelerate convergence was used to update the weights (Polyak and Juditsky, 1992). Cross-entropy loss was adopted as an optimization criterion. It is a logarithmic function that determines the “distance” between the true and estimated probability distributions (Murphy, 2012). For discrete target values, minimizing cross-entropy is equivalent to minimizing the negative logarithm probability (under the model) of the correct class. To handle class imbalance, weights for each class in the train set were calculated as one divided by the number of examples in the class and included into the cross-entropy term. This helped to avoid bias toward the majority class, a bottleneck of class imbalanced datasets.

### Training, evaluation, and revision

#### Training and evaluation

The data were split into training, validation, and test sets using subject-wise five-fold cross-validation scheme. For each fold, 20% of the subjects was taken for the test and validation sets. The remaining 80% was used for training. The validation loss was recorded after each epoch (i.e., a full training pass over all the minibatches; a full training run usually consists of several epochs) next to the train loss to examine the learning dynamics of the model. After the train-validation loop, the train and validation sets were pooled and passed through the network using the latest network’s parameters on the loop exit. The parameters were optimized one more time as one epoch was performed on the combined set. Performance metrics such as Sensitivity, Specificity, Precision, and Balanced Accuracy (*bAcc*) were recorded on the test fold at the probability threshold of 0.5 (see Eqs. 1–4). Then, the average performance was estimated across the five test folds for each metric:

(1) $$Sensitivity=\frac{TP}{TP+FN};$$

(2) $$Specificity=\frac{TN}{TN+FP};$$

(3) $$Precision=\frac{TP}{TP+FP};$$

(4) $$bAcc=\frac{Sensitivity+Specificity}{2}.$$

In the equations above, *TP* is the number of true positives, *FN* is the number of false negatives, *FP* is the number of false positives, and *TN* is the number of true negatives.

#### Revision

The final model was trained using the same hyperparameters on the entire dataset without splits over 100 epochs. The final fit to the data were then used to determine false positive and false negative EEG segments, i.e., where the model disagreed with the original annotation. The segments were independently revised by two trained experts with years of practice, and interrater agreement was evaluated using Cohen’s κ (Eq. 5). Subsequently, original annotations of EEG segments for which the two raters agreed on the new annotation were replaced with the latter. The model was then retrained on the original plus revised data according to the scheme described above.

(5) $$k=\frac{p_{o}-p_{e}}{1-p_{e}}.$$

In the equation above, $p_{o}$ is the relative observed agreement between the two raters, and $p_{e}$ is the probability of chance agreement, which for m categories and N observations is $p_{e}=\frac{1}{N^{2}}\underset{m}{\sum }n_{m1}n_{m2}$, where $n_{m1}$ and $n_{m2}$ are the number of times category m was predicted by rater 1 and 2, respectively. Cohen’s κ ranges from −1 to 1, with 1 corresponding to perfect interrater agreement and 0 corresponding to chance-level agreement. As suggested by Cohen, k of ≤0 indicates no agreement, 0.01–0.20 none to slight agreement, 0.21–0.40 fair agreement, 0.41–0.60 moderate agreement, 0.61–0.80 substantial agreement, and 0.81–1.00 almost perfect agreement.

### Data availability and code accessibility

Because of ethics and privacy regulations of human subjects, we cannot share the clinical data used for training. The code/software described in the paper is freely available online at GitHub repository (https://github.com/dmari104/CNN-EEG). The code is available as Extended Data 1.

10.1523/ENEURO.0160-22.2022.ed1 Extended Data 1 The code used to perform preprocessing of the data and all experimental work. The zip file contains pipeline scripts arranged in subfolders indicating the order of execution, a txt file with all dependencies (requirements.txt), and a short description for each step of the pipeline (README.txt). Download Extended Data 1, ZIP file.

### Software and hardware

Data were preprocessed and analyzed on an Intel(R) Core(TM) i7-8565U CPU at 1.80 GHz with 16 GB of RAM and dual four cores, running on Ubuntu 18.04.3 LTS (Bionic Beaver). Data were preprocessed using functions from *MNE Python* as well as custom-made functions. Model training was implemented in Python 3.7.6 using *PyTorch* 1.5.0 with CUDA 10.1 compatibility on the GPU-based (NVIDIA Titan/GTX980/K20/K40) Distributed ASCI Supercomputer 5 platform (Bal et al., 2016), DAS-5, using the VU cluster at 2.4 GHz with 64 GB of RAM and dual 8 cores, running on CentOS Linux 7. Re-annotation of EEG segments was performed in MATLAB R2019a (The Mathworks Inc., 2019).

## Results

### CNN learns to distinguish and generalize manually annotated EEG artifacts and nonartifacts

To assess the ability of the CNN to identify artifact and nonartifact patterns in EEG signals, a model was trained on expert-annotated artifact (*n = *50,806) and nonartifact EEG segments (*n = *125504) of 1 s each from 340 resting-state EEG recordings of 171 subjects (see Materials and Methods, Data). The expert annotations served as a gold standard to perform subject-wise five-fold cross-validation by splitting the data into train, validation, and test sets (see Materials and Methods, Training, evaluation, and revision).

**Figure 2.**
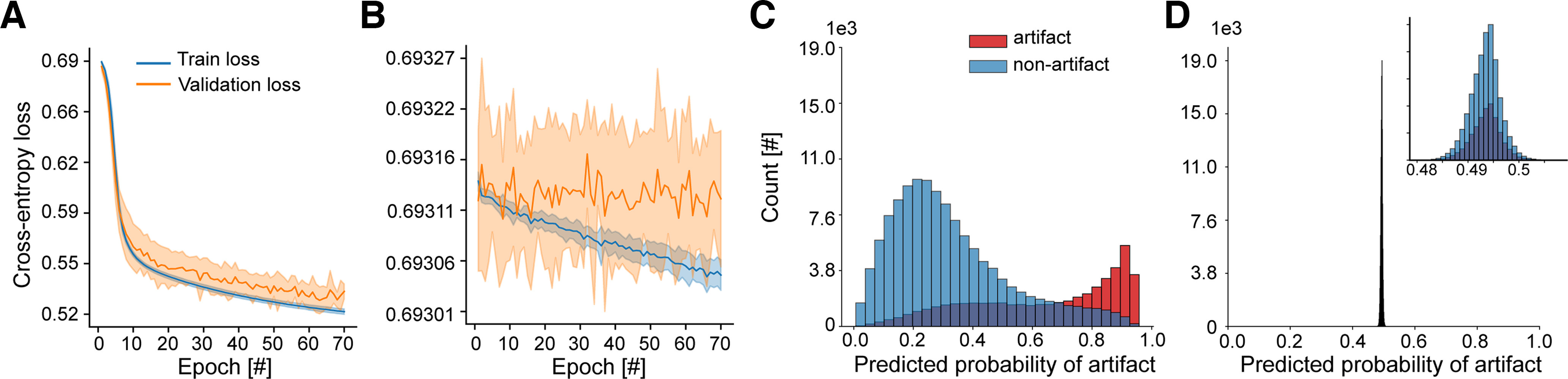
The classifier predicts manually annotated artifacts and nonartifacts with good accuracy. ***A***, Train- and validation-loss curves gradually decrease and converge as the training progresses with each training epoch for the classifier trained on expert-annotated artifacts and nonartifacts. ***B***, The classifier trains poorly on the same data but with randomly sampled annotations, showing no decrease for the validation-loss curve and overfitting to the training set as indicated by the diverging loss curves. The mean curve ± SD (shaded area around the mean curve) over five test folds is shown. Subject-wise five-fold cross-validation was used in each case. The legend and *y*-axis are shared between ***A*** and ***B***. ***C***, The classifier separates expert-annotated artifacts from nonartifacts, and most EEG segments in each class are classified confidently and correctly. ***D***, The random classifier cannot separate randomly labeled EEG segments and lacks confident predictions. The number of EEG segments is plotted on the *y*-axis, and the predicted probability that an EEG segment has an artifact on the *x*-axis. The legend and *y*-axis are shared between ***C*** and ***D***. The second distribution inside ***D*** is a zoomed-in version of the main distribution with the same *y*-axis.

During training, loss on training and validation sets were recorded, which informed how well the model fit the training and validation data, respectively. The train-validation dynamics displayed a good learning pattern with no overfitting, as indicated by the decrease in both train- and validation-loss curves and their convergence to a minimum with the increasing number of epochs (Fig. 2*A*). This contrasted with the learning pattern of the model that was trained on the same data but with annotations randomly sampled (i.e., random gold standard), which served as baseline and validity check (Fig. 2*B*). In this case, random sampling was done with class probabilities equal to the ratio of examples in each class in the original data. The average test performance of the classifier across the five folds for four different metrics is shown in Table 3 and was higher compared with that of the random case. The final model trained on the entire data for 100 epochs demonstrated good class separation as shown by the probability distribution of model predictions in Figure 2*C*. Under the used gold standard, the bulk of EEG segments were confidently assigned to their class, which contrasted the output in the random set-up (Fig. 2*D*). These findings suggested that the used gold standard contained distinct artifact and nonartifact patterns that could be learned and distinguished by the model as well as generalized across subjects, in contrast to the random gold-standard case. However, it could be seen that the separation of the two classes and generalizability of the model under the used expert annotations were not perfect. There were data of both classes within the uncertainty range of model’s confidence (0.45–0.55 probability) as well as data falsely classified with moderate to high confidence by the model ([0.0, 0.40] and [0.65, 1.0]). This raised a question of who, the gold standard or the model, was right, especially for misclassified data with moderate to high confidence.

**Table 3:** The CNN classifier shows good test performance

Model	Sensitivity %	Specificity %	Precision %	bAcc %
CNN CNN-rnd	71.0 ± 5.5 4.6 ± 3.1	78.1 ± 3.5 95.3 ± 3.3	59.7 ± 2.7 30.0 ± 3.5	74.6 ± 1.3 49.9 ± 0.2

Average test performance scores are shown for four different metrics. CNN, classifier trained on expert-annotated EEG dataset; CNN-rnd, classifier trained on the same dataset where annotations were randomly generated with class probabilities equal to the ratio of examples in each class in the original dataset; bAcc, balanced accuracy. Mean ± SD values are shown. Subject-wise five-fold cross-validation was used in each case. Scores were calculated based on the probability threshold of 0.5.

### The model uncovers artifacts misclassified by the expert annotator

**Figure 3.**
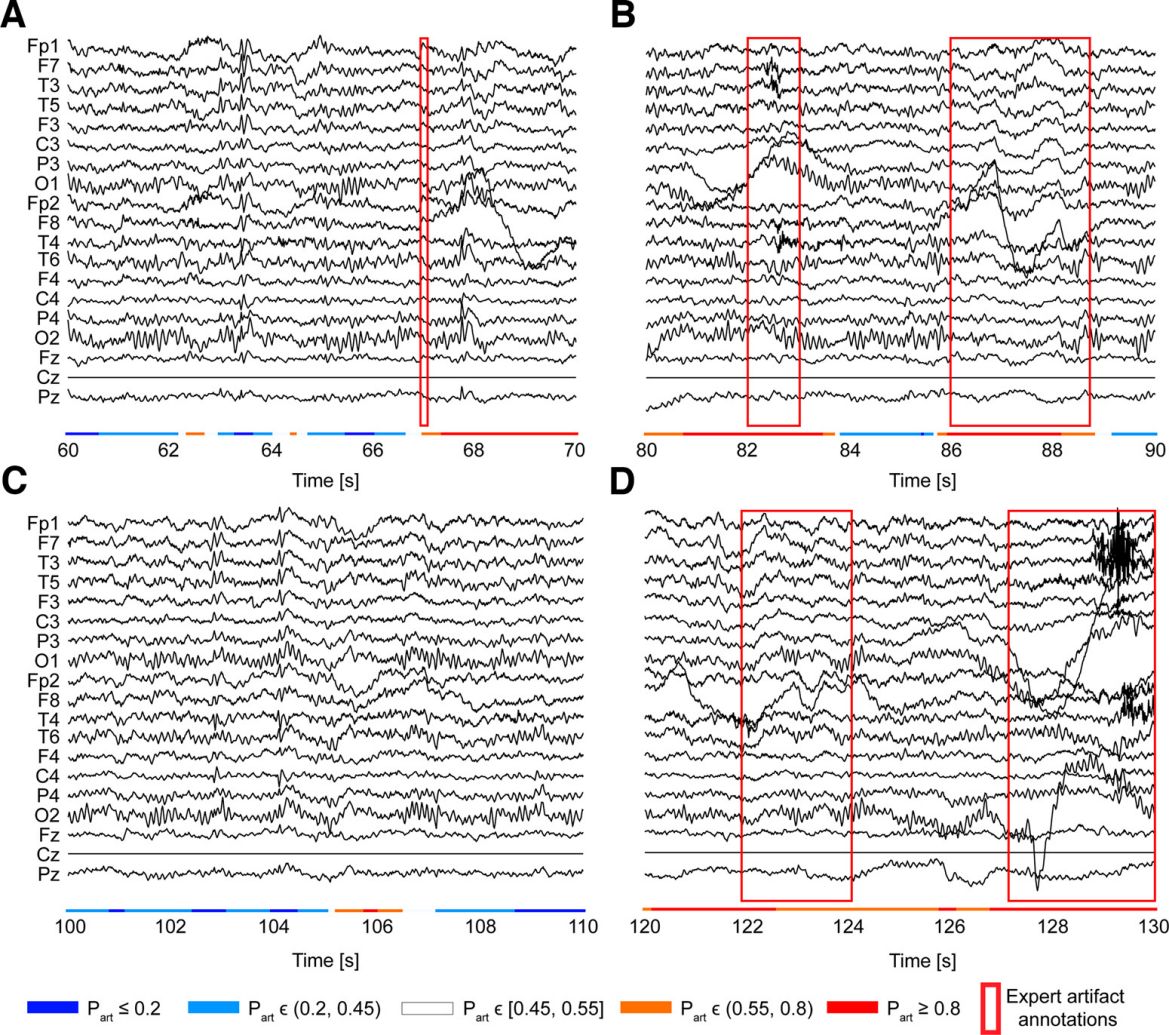
The model uncovers artifacts missed by the expert annotator. Examples of manually annotated EEG signals with corresponding model predictions from one of the recordings are shown. The model detected artifacts marked by the expert (***A***) at 67 s, (***B***) between 82 and 83 s and 86 and 89 s and (***D***) 122 and 124 s and 127 and 130 s. It also detected additional possible artifacts between (***A***) 67 and 70 s, (***B***) 80 and 82 s, (***C***) 105 and 107 s, (***D***) 120 and 122 s, and 124 and 127 s. Nineteen standard EEG channels are specified on the *y*-axis. The color bar below the signals represents probability-based predictions made by the CNN model. The color indicates one of the five categories of probability of an artifact $P_{art}$. Here, each time sample has a corresponding probability value. For that, predictions made by the model for 1-s overlapping windows (50% overlap) were interpolated for each time sample using three consecutive windows at a time (the current window, the window before, and the next window), except the first and last second of the recording for which only two consecutive windows were used for interpolation.

To facilitate interpretation of the results, we further looked at the correspondence between the model’s output and the gold standard. We examined one of the EEG recordings and compared the predictions made by the model against the annotations made by the expert. At the probability threshold of 0.5, the model made 83% of correct predictions, identifying 76% of artifacts and 86% of nonartifacts. Examples of such predictions are shown in Figure 3, where the model detected five artifact intervals marked by the annotator. More importantly, the classifier detected five more intervals (67–70 s in Fig. 3*A*, 80–82 s in Fig. 3*B*, 105–107 s in Fig. 3*C*, 120–122 and 124–127 s in Fig. 3*D*) that were not marked by the annotator [some of them probably by accident, e.g., a misclick or software malfunction (the interval between 67–70 s seems to be missed by the annotator by accident (e.g., a misclick)] but were corroborated to be artifacts. This suggested that the model could have outperformed the gold standard in some of the cases in the rest of the data. A re-assessment of such cases by two trained experts could shed light on the degree of actual correct hits made by the model. It may also improve the model by reducing the noise from misinformation and mistakes contained in the gold standard.

### The model training behavior and performance change under the expert-revised gold standard

**Figure 4.**
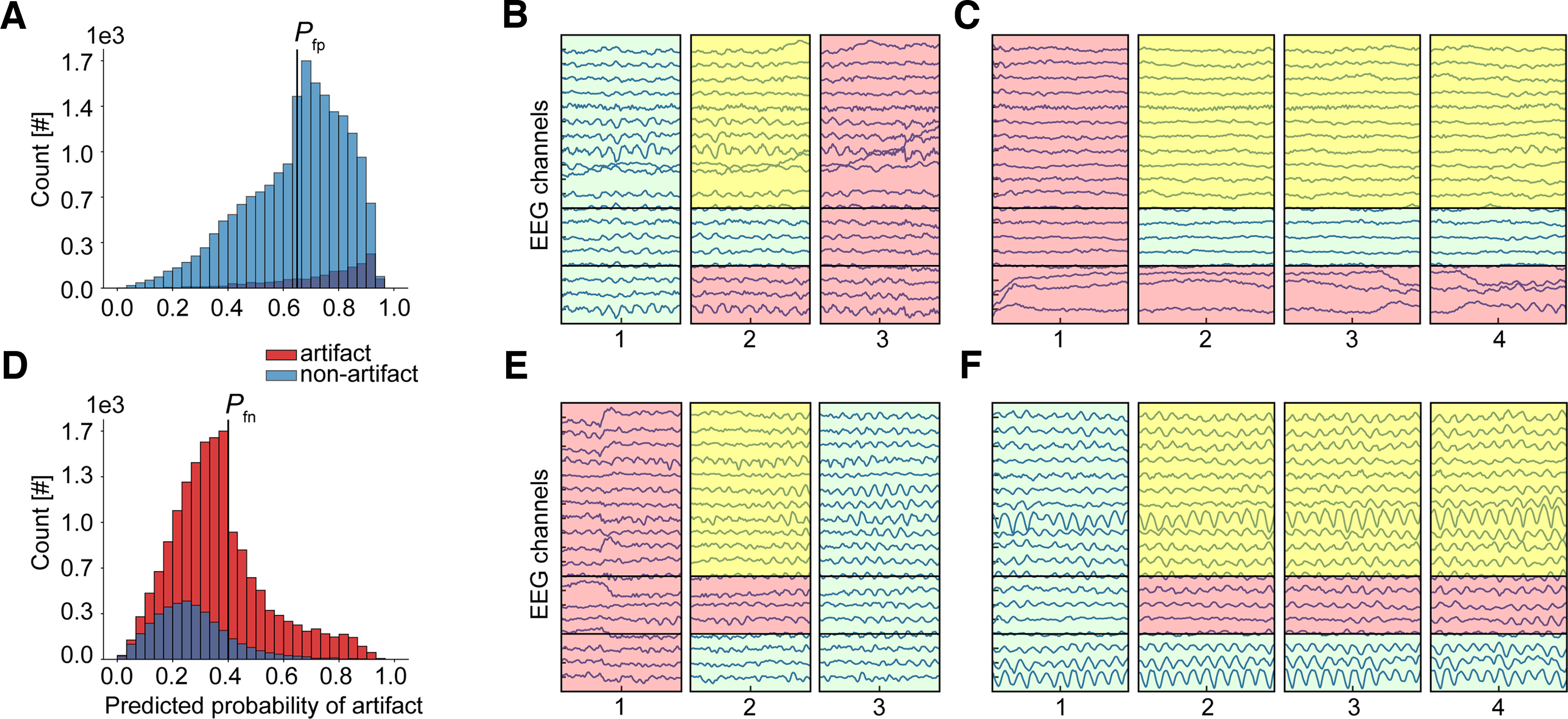
Examples of EEG segments selected for revision. Probability distributions of segments with predominantly false positive (***A***) and predominantly false negative (***D***) model predictions. Predicted probabilities that EEG segments have an artifact are depicted on the *x*-axis, and the legend shows original expert annotations. The selection was made based on the predicted probability range [0.65, 1] for ***A*** and [0, 0.4] for ***D***, including segments that were adjacent to the selected segments in time, regardless of their predicted probability value. That is why one can see segments of both classes with predicted probabilities <0.65 or >0.4. Probability thresholds, *P_fp_* and *P_fn_,* are shown on the plots as black vertical lines with the corresponding label. ***B***, ***C***, ***E***, ***F***, Examples of EEG segments selected for revision. ***B*** and ***C*** show adjacent overlapping segments with false positive (rectangle 2 in ***B*** and rectangles 2, 3, and 4 in ***C***), true positive (rectangle 3 in ***B*** and rectangle 1 in ***C***), and true negative (rectangle 1 in ***B***) predictions. ***E*** and ***F*** show adjacent overlapping segments with false negative (rectangle 2 in ***E*** and rectangles 2, 3, and 4 in ***F***), true negative (rectangle 3 in ***E*** and rectangle 1 in ***F***), and true positive (rectangle 1 in ***E***) predictions. Horizontal lines in each example that separate each rectangle in three regions show areas that were shaded according to the predicted annotation (bottom), gold standard (middle), and revised label (top). The predicted annotation was decided based on the predicted probability threshold of 0.5 (nonartifact if the threshold probability was <0.5 and artifact if ≥0.5). The larger top area was shaded by default in yellow if there was a disagreement between the predicted annotation and the gold standard, in red if there was an agreement for an artifact, and in light green for a nonartifact. In the revision process, experts were presented with such segments to re-assess and make a final decision by changing or keeping the annotation in the top area. The experts were blind to the origin of annotations reflected in the bottom and middle regions, i.e., they did not know which was the predicted annotation and which was the gold standard.

Based on the previous results that showed omissions in the used gold standard, we revised a portion of segments that were misclassified by the model. Nonartifact segments that were incorrectly classified as artifacts (false positives) with the probability of [0.65, 1], artifact segments that were incorrectly classified as nonartifacts (false negatives) with the probability of [0, 0.4], as well as any segments adjacent to those segments in time, regardless of the predicted probability, were selected to be re-assessed by two independent experts (Fig. 4*A*,*D*). The thresholds were chosen to include medium- to high-confidence predictions. The experts were blindly presented with the selected segments (40 478 segments, or 23% of all segments) and could either keep the current annotation or change it to one of the following: artifact, nonartifact, or uncertain (the latter, to avoid mistakes in cases when they were hesitant about their decision). Examples of the selected segments are shown in Figure 4*B*,*C*,*E*,*F*. An extra category (i.e., gray) was added in case experts wanted to annotate brain-related physiological activity that might be necessary to remove at later stages if only “awake” periods were to be evaluated. Examples of this included slow waves typical of drowsiness (slow δ and θ activity in the background, 1–7 Hz), and hypnagogic hypersynchrony (paroxysmal sharp, high voltage δ activity characteristic of drowsiness in children; Britton et al., 2016). This category would allow for further differentiation and flexibility.

**Figure 5.**
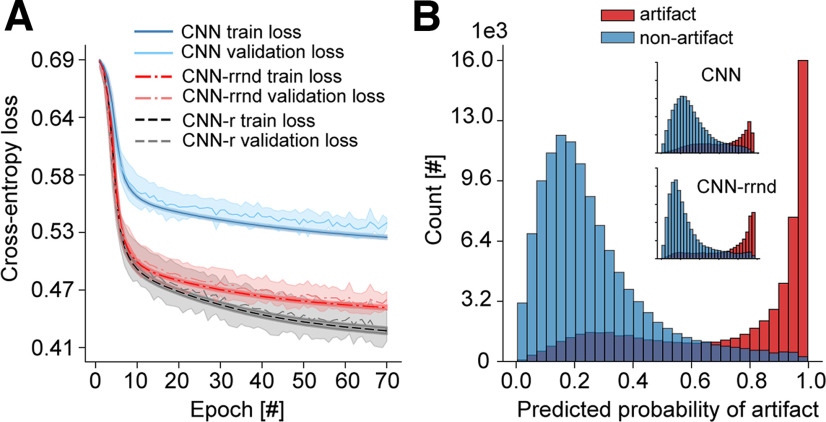
Training and performance of the CNN classifier change after expert revision. ***A***, Train- and validation-loss curves of the CNN classifier trained on the dataset with expert-revised segments (CNN-r) show improved converging dynamics as compared with the original classifier (CNN) and classifier trained on the same dataset where annotations of the revised segments were randomly generated (CNN-rrnd). The mean curve ± SD (shaded area around the mean curve) over five test folds is shown. Subject-wise fivefold cross-validation was used in each case. ***B***, CNN-r classifier shows improved separation between artifact and nonartifact EEG segments. The two probability distributions inside the plot are the distributions of the original CNN classifier (CNN) and the classifier CNN-rrnd and show changes in the distribution shape. The number of EEG segments is plotted on the *y*-axis, and the predicted probability that an EEG segment has an artifact on the *x*-axis. The two small distributions in ***B*** have the same *y*-axis and *x*-axis scales as ones of the main distribution.

The raters reached interrater agreement of 0.54 as measured by Cohen’s κ. This degree of agreement is not high and highlights the challenges and degree of subjectivity in the interpretation of subtle EEG events. Such events mostly come from the false negative portion of EEG segments, i.e., putative nonartifacts as predicted by the model (κ statistic of 0.41 vs 0.68 in the false positive portion). In total, agreement between the raters occurred in 79% of the cases (32,149 segments). Six segments were assigned to the gray class by one or both raters and were removed entirely from the dataset and subsequent computations. Segments for which the raters disagreed or which both raters assigned to the uncertain class were kept in the dataset with their original annotation. Taking this into account, annotation change occurred in 25% of the cases (10,150 segments). The dataset was then updated accordingly and contained 60,672 artifact and 115,632 nonartifact EEG segments. It was used to train and cross-validate the model from scratch. The decrease in the train- and validation-loss curves of the newly trained model was larger as compared with the original CNN classifier (Fig. 5*A*). As can be seen in Figure 5*A*, an improved training behavior was also noticeable when compared with a random-case CNN classifier which was trained on the same dataset but with annotations of the selected EEG segments randomly generated. The new CNN classifier enhanced the separation between artifacts and nonartifacts and became more confident in its predictions (Fig. 5*B*). Both the new CNN classifier and the random-case CNN classifier outperformed the original CNN classifier as shown by the average test performance across the five folds of cross-validation (Table 4). However, albeit detecting slightly less artifacts on average (73.5% vs 76.5% sensitivity), the model trained on the dataset with expert-revised EEG segments turned to be more precise and specific (76.7% vs 68.8% precision and 87.0% vs 83.4% specificity). These changes, however, should be interpreted with caution. Although the cross-validation test folds were formed using the same subject splits in each experiment, the annotations might differ because of the annotation change on revision. Since there is no perfect benchmark test set that could be used to confirm the improvements, we decided to analyze model predictions a bit further to see what could be driving these changes.

**Table 4:** The CNN classifier trained on the dataset with expert-revised EEG data shows increased test performance as compared with the original CNN classifier

Model	Sensitivity %	Specificity %	Precision %	bAcc %
CNN CNN-r CNN-rrnd	71.0 ± 5.5 73.5 ± 4.7 76.5 ± 4.2	78.1 ± 3.5 87.0 ± 2.0 83.4 ± 2.2	59.7 ± 2.7 76.7 ± 2.6 68.8 ± 2.1	74.6 ± 1.3 80.2 ± 1.7 79.9 ± 1.2

Average test performance scores are shown for four different metrics. CNN, classifier trained on the original dataset (see Fig. 2; Table 3); CNN-r, classifier trained on the dataset with expert-revised EEG segments; CNN-rrnd, classifier trained on the same dataset where annotations of the revised segments were randomly generated with uniform class probabilities; bAcc, balanced accuracy. Mean ± SD values are shown. Subject-wise five-fold cross-validation was used in each case. Scores were calculated based on the probability threshold of 0.5.

### The gold standard can be improved further

We analyzed predictions made by the old and new model on three subsets of the training data. The first subset was a portion of the revision set for which both expert decisions agreed with the original annotation (54% of the revision data, or 12% of all data). The second subset was a nonrevised portion of the data (82% of all data). The third subset was a portion of the revision set for which there was a disagreement between the original and new annotation, hence a change in the annotation by both experts (25% of the revised data, or 6% of all data).

**Figure 6.**
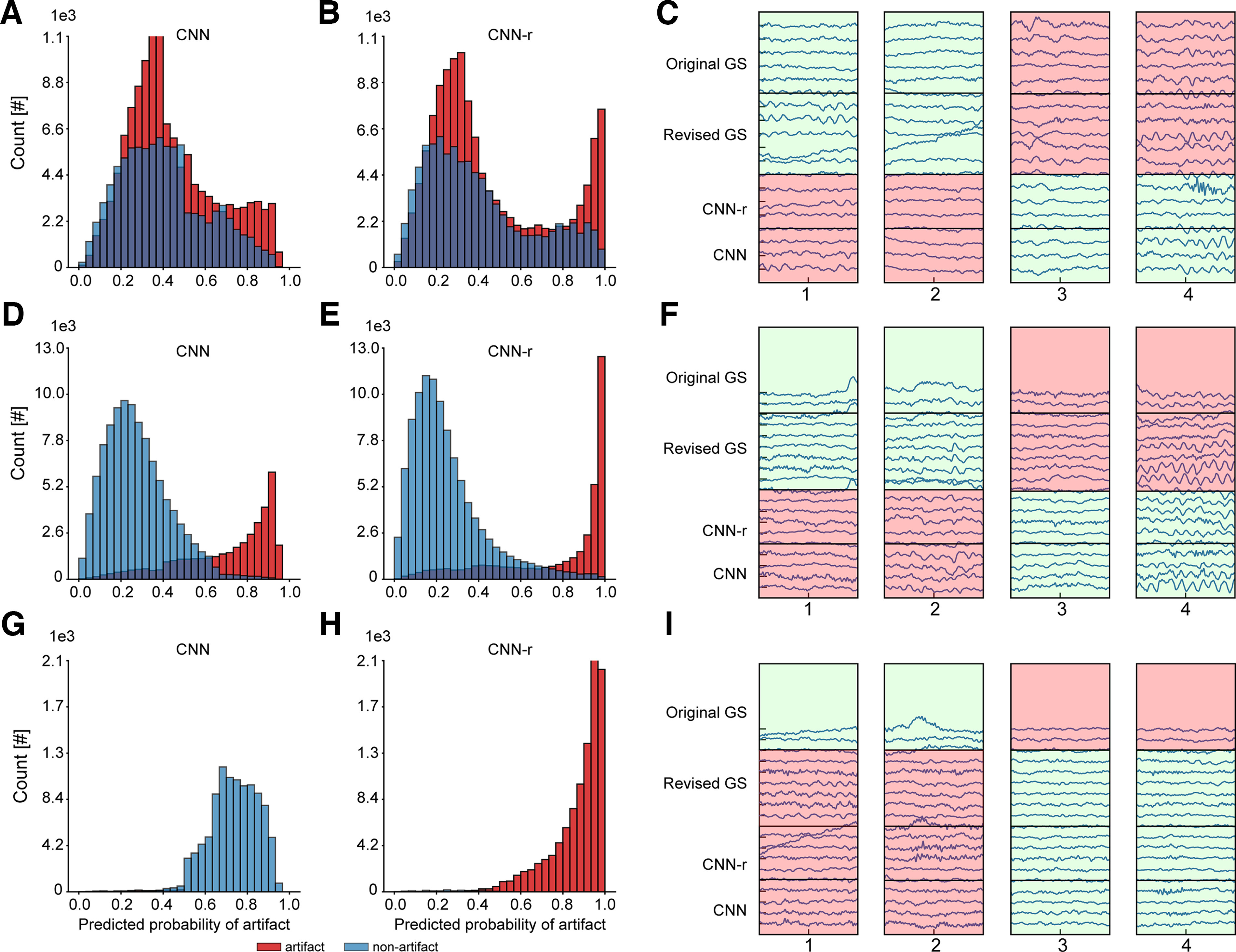
The CNN model becomes more confident in its predictions after expert revision. Distribution of the predicted artifact probabilities plotted for the subset of the revision data for which both expert decisions agreed with the original annotation shows agglomeration of values in a high-confidence range for the model trained on the dataset with expert-revised EEG segments (***B***) as compared with that of the model trained on the original dataset (***A***). ***D***, ***E***, The same trend is observed in the distributions plotted for the subset of the nonrevised portion of the data. ***G***, ***H***, Distributions are plotted for the subset of the revision data for which both experts changed the original annotation. ***G***, Under the original gold standard, the CNN model trained on the original dataset predicted most nonartifacts with high probability of being artifacts, whereas (***H***) under the expert-revised gold standard, most of these segments changed their annotation to artifacts and were predicted with high probability of being artifacts by the CNN model trained on the dataset with expert-revised data. For all plots, the number of EEG segments is plotted on the *y*-axis, and the predicted probability that an EEG segment has an artifact on the *x*-axis. ***C***, ***F***, ***I***, Examples of EEG segments for 19 EEG channels (*y*-axis) predicted by the models with original and revised annotations. Horizontal lines in each example that separate each rectangle in four regions show areas that are shaded according to the predicted annotation by the original CNN model (first from bottom), predicted annotation by the new CNN model (second from bottom), original gold standard (first from top), and revised gold standard (second from top). The predicted annotation was decided based on the predicted probability threshold of 0.5; nonartifact (in green) if the threshold probability was <0.5 and artifact (in red) if ≥0.5. CNN, classifier trained on the original dataset; CNN-r, classifier trained on the dataset with expert-revised data; Original GS, gold standard based on original expert annotations; Revised GS, gold standard based on expert-revised annotations.

Both models showed similar results on the first two subsets of the training data (Table 5). Whereas they performed generally well on the nonrevised data subset (80.2% and 79.6% sensitivity, 91.0% and 90.5% specificity, and 76.1% and 74.8% precision by the original and new CNN model, respectively), they showed poor performance on the subset of the revision set for which both experts agreed with the original annotation (34% and 33.8% sensitivity by the original and new CNN model, respectively). Despite this similarity in the performance scores, the difference between the two models could be seen in the distributions of their predicted probabilities (Fig. 6). No separation between the two classes with left-skewed histograms was observed for the original CNN model on the subset of the revision set for which both experts agreed with the original annotation (Fig. 6*A*), hence low performance in predicting artifacts (Table 5). However, despite the same low performance of the new CNN model, the predicted probability distribution showed a trend for separating the two classes with two-tailed histograms (Fig. 6*B*). Two-tailed distributions were also visible for the nonrevised subset of the data with good separation between artifacts and nonartifacts shown by both the original and new CNN model, where the latter predicted artifacts and nonartifacts more confidently (Fig. 6*D*,*E*). Nevertheless, both models showed imperfect class separation, which could indicate that both models missed artifact and nonartifact EEG patterns detected by the two experts or they identified such patterns that were missed by the experts. Examples of artifact and nonartifact signals from both subsets of the data showed that some of such events, subtle or distinct, could indeed be missed by either the models or the experts (Fig. 6*C*,*F*).

**Table 5:** Portion of the dataset revised by the two experts drives the changes in CNN training behavior and performance

Subset	Gold standard	Model	Sensitivity %	Specificity %	Precision %	bAcc %
R-a	Original ( =Revised)	CNN	34.0	72.8	63.5	53.4
	Revised ( =Original)	CNN-r	33.8	71.4	62.1	52.6
nR-a	Original ( =Revised)	CNN	80.2	91.0	76.1	85.6
	Revised ( =Original)	CNN-r	79.6	90.5	74.8	85.0
R-d	Original ( ≠Revised)	CNN	0.0	1.6	0.0	0.8
	Revised ( ≠Original)	CNN-r	98.6	97.2	100	97.9

R-a, subset of the revision data for which both expert decisions agreed with the original annotation; nR-a, nonrevised portion of the data; R-d, portion of the revised set for which there was a disagreement between the original and revised annotation; Original, gold standard based on the original expert-annotated dataset; Revised, gold standard based on the dataset with expert-revised EEG segments; Original = Revised, in that subset of the data, the two gold standards agree; Original≠Revised: the two gold standards disagree in that subset of the data; CNN, classifier trained on the original expert-annotated dataset; CNN-r, classifier trained on the dataset with expert-revised EEG segments; bAcc, balanced accuracy. The scores were computed based on the probability threshold of 0.5.

A well-defined difference between the two models was observed on the third subset of the data, a portion of the revision set for which there was a disagreement between the original and revised annotation. The original model showed poor results as opposed to the new model (0.0% and 98.6% sensitivity, 1.6% and 97.2% specificity, and 0.0% and 100% precision by the original and new model, respectively; Table 5). This was expected as the portion of the data to be revised was determined based on the false predictions made by the original CNN under the original expert-annotation gold standard. As can be seen from Figure 6*G*, the false predictions in the subset of the data for which the original annotations were later changed by the two experts were mostly the false positives (i.e., putative artifacts). In total, originally 10,008 nonartifacts and 142 artifacts changed their annotation, and later most of those cases were confidently and correctly predicted by the new CNN model under the expert-revised gold standard (Fig. 6*H*,*I*). Based on these results, the revised portion of the data brought about the changes in the CNN model training behavior and performance, and the new model became generally more confident in its predictions. It also showed that the gold standard could be improved further.

## Discussion

Resting-state EEG is commonly used by researchers and clinicians to analyze intrinsic brain activity and compute biomarkers of various developmental and mental health disorders. Analysis outcomes depend on the quality of upstream cleaning and preprocessing, which are typically performed by trained professionals who visually inspect and manually annotate EEG signals. Interpretation of EEG patterns can be extremely challenging, time consuming, and flawed. In this paper, we presented a CNN to increase the speed and accuracy of manual annotation of artifacts in resting-state multichannel EEG recordings. Our findings demonstrate that the model is capable of learning artifact and nonartifact patterns in manually annotated EEG signals and converging with a better gold standard. Re-assessment of controversial EEG patterns, i.e., those that CNN confidently predicted as artifacts or nonartifacts in disagreement with the experts improved both the model and the ground truth. The experts changed labels in 25% of the selected segments, which led to improved performance of the revised model (CNN-r) and supported our hypothesis. In the control experiment where the labels of the selected segments were randomly shuffled, CNN-rrnd performed worse than CNN-r, as expected. Although it might seem to be counterintuitive at first that the performance of CNN-rrnd was better than that of CNN, this was expected because most of the data were the same in the two models, but when randomly shuffling the labels of the selected data, by mere chance, some would flip and match the new expert-revised annotation, which would lead to enhanced performance of CNN-rrnd as compared with that of CNN which operated under the original uncorrected ground truth.

We envision that our approach may operate semi-automatically in the future and be particularly useful for helping annotators in their daily work, especially when processing large datasets with a high degree of artifact contamination. The model can be applied for automatic annotation of patterns predicted with certain confidence. The annotator will then only have to score portions of the signal predicted with low reliability, which thereby will reduce the amount of data left to be examined and scored by the annotator. It may also be interesting to test how our method works in combination with other cleaning approaches such as HAPPE, ADJUST, or FASTER (Nolan et al., 2010; Mognon et al., 2011; Gabard-Durnam et al., 2018). For example, our model can be used as the next step of the pipeline to make predictions on the data coming out of these approaches to let the annotator inspect segments indicated by the model with high confidence to be artifacts. This may be a way of evaluating upstream cleaning and leaving room for further cleaning without having to manually inspect a large number of segments already properly cleaned by these algorithms. By integrating our method into a signal viewer that is currently being designed in our group (Weiler et al., 2022), we expect to facilitate fast and reliable resting-state EEG data analyses.

Several evolving large-scale EEG-data curations (Harati et al., 2014; Cavanagh et al., 2017) are a result of exceptional effort and time that are being put into collecting, organizing, and realizing data which continue to support the development and testing of various machine-learning algorithms. Nevertheless, it still is hard to design advanced versatile approaches for all-purpose EEG-pattern recognition or faithfully compare existing detection algorithms. Partly, this is because of still inadequate quantities or heterogeneity of properly annotated ground-truth data, and partly, because massive number of EEGs remains private or unannotated. Some methods, thus, may work better than others for one type of data and vice versa for another type of data. We acknowledge that our model is no different as based on a limited set of data and confined to certain conditions and experimental setting. Professional judgment by trained medical experts is ultimately indispensable to ensure the quality and validity of decisions and performed analyses, and the model should be used as a decision-support system. To the best of our knowledge, there is no properly annotated resting-state neurodevelopmental EEG-data curation accessible to public. We hope making our model available for other labs with similar data to use it and, whenever ethically possible, making the data we annotate accessible to public. We should note that the clinical measurements used in our study were obtained from children aged 7–16 years when EEG is not entirely mature. Indeed, EEG patterns of brain activity evolve with age [e.g., the posterior dominant rhythm evolves to an α one (8–12 Hz) by age 5–13 years, sleep patterns become fully developed in school-aged children, and specific EEG patterns are more prominent, such as λ waves, positive occipital sharp transients of sleep, and hypnagogic hypersynchrony; Britton et al., 2016]. However, the nature of most EEG artifacts does not evolve over time (e.g., eye blinks, eye movements, pulse and muscle activity, or nonphysiological artifacts). Thus, we expect the model to perform well also in an adult population, as with the mean age of 10 years, the signal comes rather close to an adult EEG. This will be further tested in upcoming studies.

As more data get curated by human experts, we highlight the feasibility to iteratively improving our model through active learning. Similar work was done by Yang and colleagues (S Yang et al., 2017) where the authors used self-training to improve detection performance in clinical EEGs. They did initial training on a small set of labeled data and used the model to automatically annotate unlabeled events with high-confidence scores to include them in the next training iteration, repeating the last two steps until all unlabeled data got annotated. Thus, expert intervention was eliminated. Our approach, on the other hand, needs human intervention. We argue that it is important to ensure that the model is being exposed to EEG patterns in which it is least confident, and which are probably the most subtle and informative. Resolving such cases by experts would secure feature variability and veracity in the gold standard. As we see, human error and subjectivity in making decisions are inevitable, thus we should aim at enhancing the interrater agreement when revising EEG segments. This can be done by letting experts revise the data for the second time together, providing a possibility to discuss and arrive at a final decision. It may also be useful to turn to multi-class classification and stratify EEG patterns into distinct categories, as is being done in several corpora (Harati et al., 2015; Buckwalter et al., 2021). This can include separate categories for different types of ocular and muscle artifacts (e.g., blinking, lateral eye movement, eye flutter, glossokinetic, and chewing), as well as various abnormal brain-related EEG patterns (e.g., slowing of activity, sharp waves, spike-wave complexes, etc.). The latter might be particularly useful when analyzing datasets where EEG abnormalities are highly prevalent (e.g., EP and neurodevelopmental disorders) possibly discerning between signs of a more generalized cortical dysfunction from localized epileptiform abnormalities (Bruining et al., 2020). This way, it would be possible to vary the definition of artifacts depending on the task at hand as well as help annotators spot physiological artifact-free signals of interest. We consider these improvements for future work.

We also note the lack of statistical tests as one of the current limitations. Statistical testing using repeated five-fold cross-validation at both experimental stages (before and after revision) would strengthen the conclusions of our analysis but would be very demanding to realize, considering computation costs associated with training a model on a single fold (∼20 h for 70 epochs of training, which would add up to 2000 h for 10 runs of the two five-fold cross-validation experiments each). This is excluding intrarater reliability testing for each rater in the manual revision step of our pipeline which would be even more challenging to implement as single re-annotation of 25% of the data takes five full days of work.

In the short-term, we aim at developing a signal viewer that would allow using our approach as a decision-support and guidance system for manual or semi-automatic annotation of artifacts in resting-state EEG recordings. It would also allow us to accumulate more labeled data, re-train the model, and run the next iteration to improve the gold standard.

## Synthesis

Reviewing Editor: Niraj Desai, National Institute of Neurological Disorders and Stroke Decisions are customarily a result of the Reviewing Editor and the peer reviewers coming together and discussing their recommendations until a consensus is reached. When revisions are invited, a fact-based synthesis statement explaining their decision and outlining what is needed to prepare a revision will be listed below. The following reviewer(s) agreed to reveal their identity: Tagio Falk, Ruggero Scorcioni.

First, let me apologize for how long the review process on this manuscript has taken. Given the study’s interdisciplinary nature, it was challenging to find qualified reviewers.

But now, two very good reviewers have read the manuscript, as have I. All of us were impressed by your work and think the study will contribute importantly to the literature on EEG/ML. Both reviewers had ideas for improving and clarifying the manuscript, which we think can be addressed and/or implemented in short order. These are described in the reviewer reports that I have appended. When you resubmit, please address these ideas point by point.

I thank you for submitting this manuscript to eNeuro, and, again, I apologize for how much time this review process has consumed.

REVIEWER #1

I really enjoyed reading this article and liked the idea of building a tool to help clinicians. The paper is very solid, very well written and has done several experiments to back up the claims. I do have some concern about the lack of statistical tests (see note below) but realize that for work like this one, it can be very challenging and time consuming. So I suggest the authors mention it as a “study limitation”. Also, it would be nice to have some motivation as to why a CNN was used. There are many other more “temporal friendly” architectures (eg, RNN, LSTM, biLSTM, etc) that could have been taken advantage of the time-domain properties of many artifacts. Was there a specific reason for sticking to CNNs?

Also, I may have missed this part. But after the first training, the test set is evaluated and this is where the two raters com in to fix the labels. The corrected labels (from the test set) and then mentioned to be used to retrain the model. How did this happen, exactly, as the test set data is “unseen” in training. Did the whole set get recombined and re-shuffled into 5 new cross-validation partitions for the sedonc training stage?

There are also some questions about the processing itself. It is argued that computational costs were considered when looking only at 19 channels as opposed to 64. Along these lines, the data was bandpass filtered to 45Hz; why keep the original sampling rate of 2048Hz after this pre-processing? Downsampling would incur a lot of computational cost rediction, no? Also computing the wavelet coefficients up to 2048 after this bandpass filtering would mean many of the wavelet coefficient would be not very useful, no? Also, while I do appreciate that a specific dataset was used as it comprised some human rater artifact labels, the EEG data is for kids. Given the changes observed in EEG with aging, how reliable would the models be with adults, for example? This could also be emphasized as a study limitation.

On page 7, it is mentioned that “Signals were scrolled through in windows of 10 seconds” but throughout the text, 1s segments are used. Is the 10 a typo?

Please check the punctuation after each equation and treat them as text (ie, add periods, commas, as applicable)

With your minimal pre-processing step, this is already done by many as a means of artifact removal. Since you have labels, it could be interetsing to maybe report the effects that such pre-processing has on actual artifact removal. Lastly, it could be interesting as future work to maybe run your data through an artifact removal pipeline, such as HAPPE, and then trhough your model and see what effects pre-enhancement may have on your CNN outputs. Several recent works have shown that concatenating speech enhancement methods can be helpful (HAPPE itself is a combination of methods).

Overall, a very nice paper and method. Congratulations to the authors.

NOTE ON STATISTICS: I mention here not applicable, but statistical tests could be done if the 5-fold x-validation was repeated several runs. I do realize this would be very cumbersome given the two rater step for validation after each iteration. Perhaps a line in the discussion about “study” limitations could be useful to mention this aspect.

REVIEWER #2

Concern:

1: I would expect the model performance for CNN-rrnd to be worse than both CNN and CNN-r given that (a) this model has random labels in the training and (b) these labels are crucial for training as shown by CNN-r best model’s performance. In contrast Fig 5A shows how CNN-rrnd has performance in between CNN-r and CNN. I would suggest the authors to: (a) train multiple CNN-rrnd models with different randomization and (b) create a new model CNN-flip where all the re-labeled data are opposite to examiners’ choices. I would expect the updated Fig 5A to have, in order to worse to best model performance: CNN-flip, CNN-rrnd, CNN, CNN-r.

2: I would like the authors to update the literature review to include more articles that address EEG artifacts detection and/or removal using ML techniques to 2021/2022

Minors:

Figure 5A is difficult to read given how similar are different set of lines across multiple models. Please edit accordingly to improve readability. For example by using different line thicknesses/patterns.
